# Prediction of Hyperinflammatory Phenotypes in Critically Ill Patients via Routine Clinical Data and IL-6: Towards Personalized Anti-Inflammatory Therapy

**DOI:** 10.3390/ijms26209967

**Published:** 2025-10-13

**Authors:** Charlotte Linz, Alexander Shimabukuro-Vornhagen, Nina Hesse, Lucie Probst, Jorge Garcia Borrega, Dennis A. Eichenauer, Matthias Kochanek, Michael von Bergwelt-Baildon, Boris Böll

**Affiliations:** 1 Department I of Internal Medicine, Faculty of Medicine and University Hospital Cologne, University of Cologne, 50937 Cologne, Germany; charlotte.linz@web.de (C.L.); alexander.shimabukuro-vornhagen@uk-koeln.de (A.S.-V.); hesse_nina@hotmail.com (N.H.); lucie.probst@uk-koeln.de (L.P.); jorge.garcia-borrega@uk-koeln.de (J.G.B.); dennis.eichenauer@uk-koeln.de (D.A.E.); matthias.kochanek@uk-koeln.de (M.K.); 2 Center for Integrated Oncology Aachen-Bonn-Cologne-Düsseldorf, University Hospital Cologne, 50937 Cologne, Germany; 3 Department of Internal Medicine III, Ludwig Maximilians University, 80539 Munich, Germany; michael.bergwelt@med.uni-muenchen.de

**Keywords:** interleukin-6, cancer, neutropenia, inflammation, sepsis, cytokine storm

## Abstract

Interleukin-6 (IL-6) is a central mediator of systemic inflammation and is markedly elevated in critical illnesses, including sepsis, acute respiratory distress syndrome, and hyperinflammatory syndromes. Patient responses to immunomodulatory therapies vary, highlighting the need to better understand IL-6 regulation and its clinical implications. We retrospectively analyzed consecutive patients admitted to a medical intensive care unit in a quaternary academic center with a comprehensive cancer program, extracting clinical and laboratory data, including inflammatory markers and plasma IL-6 levels. Plasma IL-6 concentrations were measured using an electrochemiluminescence immunoassay. Survival analyses, multivariable adaptive Lasso regression, Bayesian logistic regression, and latent class analysis were performed to define determinants of IL-6 regulation, mortality, and inflammatory phenotypes. IL-6 levels were substantially elevated in sepsis (median 1150 pg/mL) and neutropenia (median 7866 pg/mL), with extreme concentrations exceeding 20,000 pg/mL when both were present. Although IL-6 across its full range was not independently predictive of intensive care unit mortality, dichotomized thresholds (≥200 pg/mL) correlated with lower survival. Advanced modeling defined a hyperinflammatory phenotype characterized by IL-6 ≥ 100 pg/mL and predicted mortality >40%, showing mortality of 58%, alongside distinct latent subgroups with heterogeneous inflammatory activity and outcomes. These results emphasize the prominent role of sepsis and neutropenia in driving IL-6 elevations and reveal inflammatory phenotypes with potential for risk stratification and targeted anti-cytokine therapy in critical illness.

## 1. Introduction

Inflammation constitutes a hallmark of critical illness, and is frequently associated with conditions such as infection and sepsis, cancer, trauma and acute respiratory distress syndrome (ARDS) [1]. Moreover, previously underappreciated hyperinflammatory syndromes, including hemophagocytic lymphohistiocytosis (HLH) and cytokine release syndrome (CRS) or immune-related adverse events, have recently gained attention since anti-inflammatory therapy targeting specific cytokines such as interleukin-6 (IL-6) and IL-1 is effective in treating these otherwise often lethal conditions [2,3]. Hyperinflammatory conditions and specific inflammatory phenotypes are associated with excessive patient mortality, regardless of the underlying etiology, and large-scale analyses have identified distinct inflammatory subtypes with potential therapeutic implications. On a much larger scale, the use of corticosteroids has been associated with improved survival in COVID-19, ARDS, and septic shock. However, the mortality benefit of anti-inflammatory treatments remains heterogeneous, implying that patients with different inflammatory phenotypes respond differently to anti-inflammatory treatment. In addition, the identification of specific inflammatory subtypes is complicated by the need for complex laboratory assessments, which are often time-consuming and not feasible for routine clinical decision-making [4,5]. The role of currently available routine inflammatory markers, such as C-reactive protein (CRP), is limited given their lack of specificity [6]. Similarly, single cytokines and chemokines, including IL-6, exhibit pleiotropic actions and complex biology. IL-6 can be produced by a variety of cell types, including endothelial cells, macrophages, and T lymphocytes, and its receptor is expressed on hepatocytes as well as certain immune cells, such as B cells, monocytes, and neutrophils. IL-6 plays a pivotal role in the regulation of systemic inflammation and is integral to the host response to acute infection. Consequently, elevated IL-6 levels are observed in bacterial, viral, and fungal infections. Beyond its role in acute infections, dysregulation of IL-6 signaling has been implicated in the pathogenesis of inflammatory and autoimmune diseases [7,8]. Conditions such as sepsis, HLH, and CRS, especially in the context of chimeric antigen receptor (CAR) T-cell therapy, are typically characterized by markedly elevated IL-6 levels [2]. IL-6 is also involved in chronic low-grade inflammatory states associated with non-communicable diseases, including atherosclerosis [9], type 1 [10] and 2 diabetes [11], osteoporosis [12], Alzheimer’s disease [13], and cancer [14].

Given the central role of IL-6 in inflammatory processes, therapeutic targeting of IL-6 signaling has been explored in various diseases. Randomized clinical trials have demonstrated the efficacy of IL-6 blockade with the humanized monoclonal antibody tocilizumab, which is now approved for use in rheumatoid arthritis, giant cell arteritis, and CRS [2]. Numerous ongoing trials are evaluating IL-6 blockade in additional clinical contexts [15,16].

Therefore, in order to identify potential candidates for anti-cytokine therapy, we investigated the association between clinical and laboratory parameters—with a particular focus on IL-6 plasma levels—in a cohort of critically ill patients. Furthermore, we employed advanced analytical methods to uncover latent inflammatory phenotypes within the cohort and to identify patients with excessive mortality and distinct inflammatory features that may be clinically recognizable.

## 2. Results

### 2.1. Patient Characteristics, Survival Analysis, and Multivariate Analysis on Mortality

We included 160 critically ill patients, as displayed in Table 1. The median age was 59 years (interquartile range (IQR) 44–69 years), and 33% were female. Given the center’s specialization, the cohort was enriched for cancer patients (44% overall, 33% hematologic malignancies); 56% had no current or prior history of cancer (“other” in the table). Overall, 43% of the patients had sepsis. In the total cohort, more than half of the patients were mechanically ventilated (53%), and 49% required vasopressors. Neutropenia was present in 19% of patients, and about one in four patients (25%) had immunosuppression, including a minority of patients (16%) who had received prior autologous or allogeneic stem cell transplant.

**Figure 1:**
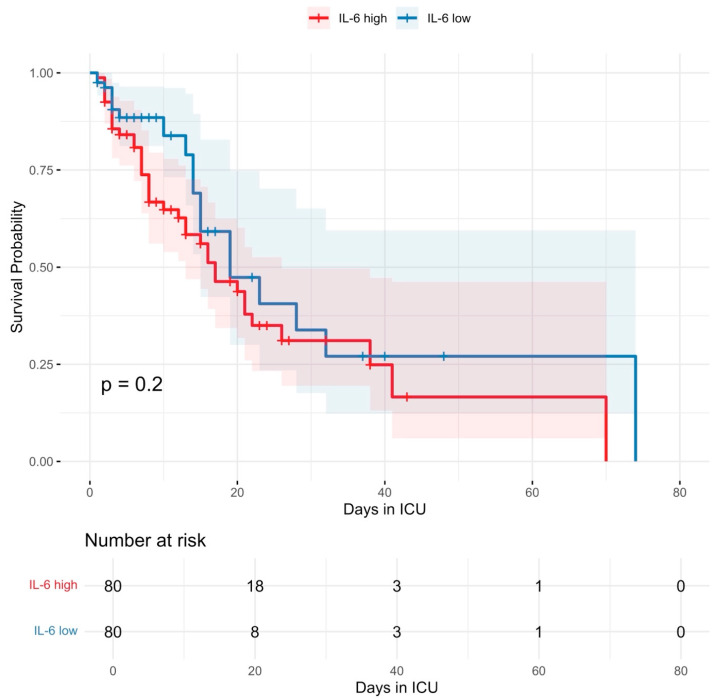
Kaplan‒Meier estimates of survival stratified by level of plasma IL-6 on day 1 of ICU (IL-6 high = IL-6 ≥ 200 pg/mL, IL-6 low = IL-6 < 200 pg/mL).

The median duration of intensive care unit (ICU) stay was five days (IQR 3–14 days), and survival until discharge from ICU was 63%. According to the Kaplan–Meier analysis, the median ICU survival (overall survival) was 19 days in the overall cohort (95% confidence interval (CI) 15–26 days), and not statistically different between patients with high (≥200 pg/mL) or low (<200 pg/mL) levels of IL-6 or diagnosis of sepsis (Figure 1). In Cox proportional hazard analysis, there was no significant association of risk of death and age, male sex, and inflammatory markers as IL-6 level or procalcitonin (PCT). In multivariate analysis with stepwise logistic regression, factors associated with overall mortality with frequentist statistics were mechanical ventilation (odds ratio (OR) = 8.78, 95% CI = 3.68–22.5, *p* < 0.001) and the Simplified Acute Physiology Score II (SAPS) score (OR = 1.92 for each point increase, 95% CI = 1.23–3.06, *p* = 0.005).

### 2.2. Association of IL-6 Plasma Levels with Clinical Variables and Neutropenia

**Figure 2:**
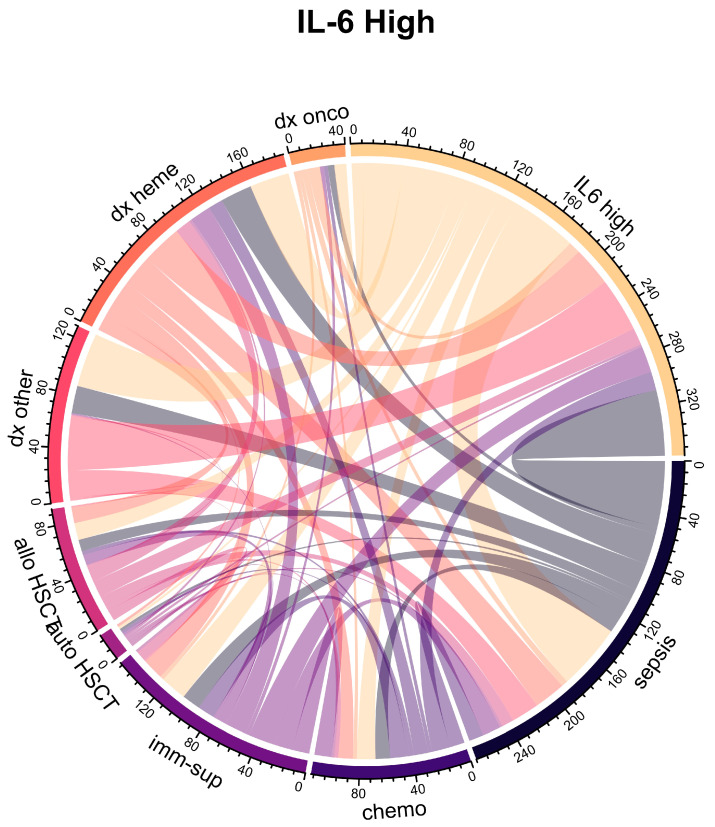

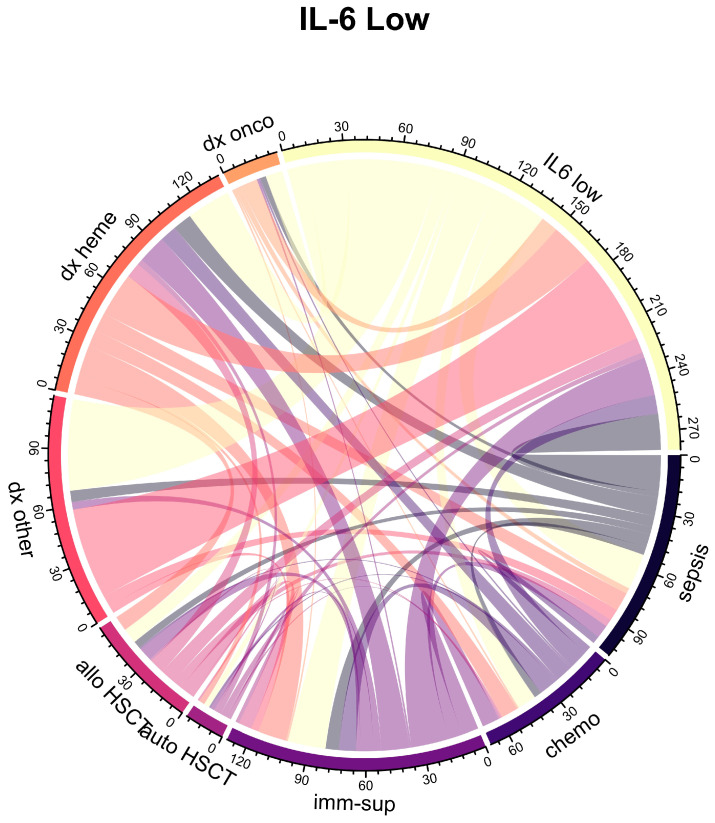
Pairwise co-occurrence of patient characteristics stratified by plasma IL-6 level on day 1 of ICU (left panel: IL-6 high = IL-6 ≥ 200 pg/mL; right panel: IL-6 low = IL-6 < 200 pg/mL).

**Figure 3:**
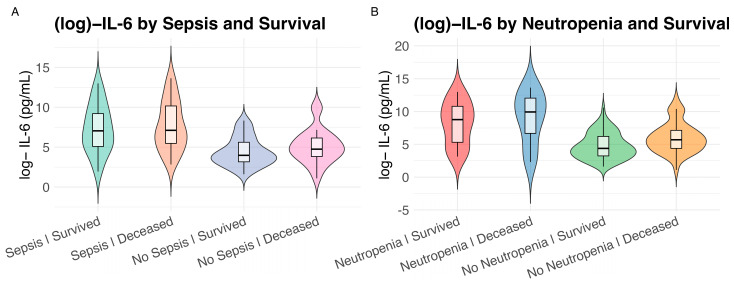
Pairwise co-occurrence of patient characteristics by plasma IL-6 level on day 1 (IL-6 high = IL-6 ≥ 200 pg/mL, IL-6 low = IL-6 < 200 pg/mL; log10 transformation was applied to reduce skewness and improve interpretability). Violin plots with boxplots represent (log10)IL-6 (pg/mL) levels by clinical subgroups. Pairwise group comparisons were analyzed using the Wilcoxon rank-sum test, significance annotated according to adjusted *p* value thresholds: (**A**) Sepsis and survival outcome. Wilcoxon rank-sum test: Sepsis|Survived vs. Sepsis|Deceased: *p* = 7.23 × 10^−1^; Sepsis|Survived vs. No Sepsis|Survived: *p* = 1.67 × 10^−6^; Sepsis|Survived vs. No Sepsis|Deceased: *p* = 5.14 × 10^−3^; Sepsis|Deceased vs. No Sepsis|Survived: *p* = 7.55 × 10^−8^; Sepsis|Deceased vs. No Sepsis|Deceased: *p* = 1.04 × 10^−3^; No Sepsis|Survived vs. No Sepsis|Deceased: *p* = 1.15 × 10^−1^. (**B**) Neutropenia and survival outcome. Wilcoxon rank-sum test: Neutropenia|Survived vs. Neutropenia|Deceased: *p* = 3.89 × 10^−1^; Neutropenia|Survived vs. No Neutropenia|Survived: *p* = 8.29 × 10^−5^; Neutropenia|Survived vs. No Neutropenia|Deceased: *p* = 1.79 × 10^−2^; Neutropenia|Deceased vs. No Neutropenia|Survived: *p* = 3.78 × 10^−4^; Neutropenia|Deceased vs. No Neutropenia|Deceased: *p* = 1.02 × 10^−2^; No Neutropenia|Survived vs. No Neutropenia|Deceased: *p* = 1.02 × 10^−2^.

The median IL-6 plasma level on the first day of admission to the ICU was 200 pg/mL (IQR 43-1576 pg/mL). Categorizing patients as either “IL-6 high” (≥200 pg/mL) or “IL-6 low” (<200 pg/mL), several characteristics differed between the two groups (Figure 2). High IL-6 levels were associated with higher prevalence of fever, need for vasopressor therapy, and lower ICU survival (*p* = 0.002) (Table 1, Figure 1). The median IL-6 plasma concentration was 117 pg/mL (IQR 29–911 pg/mL) in surviving patients (*n* = 101) compared to 400 pg/mL (IQR 87–4225 pg/mL) in patients who did not survive (*n* = 59). Not surprisingly, IL-6 levels in patients with sepsis were significantly higher than in non-septic patients (*p* < 0.0001): the median IL-6 plasma concentration was 1150 pg/mL (IQR 184-20,659 pg/mL) in patients with sepsis (*n* = 69) compared to 69 pg/mL (IQR 26–285 pg/mL) in patients without sepsis (*n* = 91) and slightly higher in deceased patients (Figure 3).

In multivariate Lasso regression analysis, mechanical ventilation, higher SAPS, higher lactate, higher CRP, and lower neutrophil count were independently associated with IL-6 levels (Table 2). Interestingly, neutropenic patients had particularly high IL-6 plasma concentrations (median IL-6 plasma concentration 7866 pg/mL (IQR 333-62,835 pg/mL, *n* = 31) and 130 (IQR 35–684 pg/mL, *n* = 129) in patients with neutropenia and without neutropenia, respectively). Although neutropenia was more likely in patients with a hematologic diagnosis, previous chemotherapy, and allogeneic hematopoietic stem cell transplantation (HSCT), none of these factors other than neutropenia itself was independently associated with higher IL-6 levels (Table 2 and Table S1).

**Figure 4:**
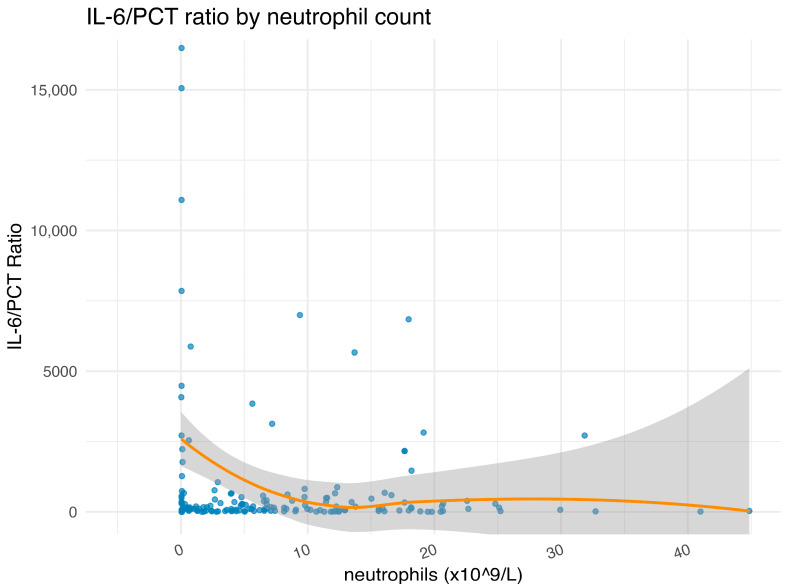
IL-6/PCT ratio versus neutrophil count (×10^9^/L) per individual patient. Each point represents one patient measurement, the smooth orange line represents a locally estimated scatterplot smoothing (loess) fit, with a shaded area indicating the 95% confidence interval. *Y*-axis truncated at 16,000 for better visibility; no formal hypothesis test is displayed on this plot.

Neutropenia, in turn, was associated with sepsis, higher inflammatory markers (CRP, PCT, and cortisol), and higher disease severity (SAPS) (Table S1). While overall survival did not differ significantly between patients with and without neutropenia, IL-6 concentrations were consistently higher in neutropenic patients irrespective of survival status, with significant pairwise differences observed across subgroups (Figure 3). IL-6 levels in neutropenic patients experiencing a strong inflammatory stimulus, i.e., sepsis, were 20,659 pg/mL (IQR 4225–170,288) compared to 533 pg/mL (IQR 140–1978) in non-neutropenic patients. Like IL-6, other laboratory correlates of inflammation, such as CRP (*p* = 0.0004), PCT (*p* = 0.0003), were significantly higher in neutropenic patients than in non-neutropenic patients (Table S1). There was a constant relationship between IL-6 and PCT across most patients. Interestingly, in neutropenic patients, there was a disproportionate increase in IL-6 with PCT, suggesting a differential regulation of the inflammatory response in neutropenic and non-neutropenic patients (Figure 4). Consequently, neutrophil count was associated with lower (log-transformed) IL-6 levels in multivariate lasso regression (Table 2), while the IL-6/PCT ratio was markedly elevated at low neutrophil counts (Figure 4).

### 2.3. Bayesian Logistic Regression and Analysis of Inflammatory Phenotypes

Two distinct groups of patients were identified using Bayesian logistic regression analysis to differentiate patients who might benefit from targeted anti-inflammatory treatment. After including all candidate variables in the model and applying posterior predictive checks for model calibration, assessing model fit, mortality probability was most accurately predicted using the variables sex, age, IL-6-level on day 1 (pg/mL), neutropenia, fever on day 1, and mechanical ventilation as predictors (Table 3). In the final model, mechanical ventilation was strongly associated with increased odds of mortality (OR = 7.25, 95% CrI: 3.14–17.00), while the other predictors had only a moderate association with mortality with wide credible intervals. Interestingly, neutropenia (yes vs. no) had a possible protective, but uncertain association (OR = 0.74, 95% CrI: 0.26–2.14).

Based on the predicted risk of mortality from the Bayesian logistic regression analysis, patients were categorized according to their inflammatory phenotype. Those with a hyperinflammatory phenotype were identified as individuals exhibiting both a high inflammatory burden, indicated by an IL-6 level greater than or equal to 100 pg/mL, and severe disease, reflected by a predicted mortality probability exceeding 40%. The IL-6 threshold of ≥100 pg/mL was selected based on a sensitivity distribution analysis and practical considerations for rapid clinical decision-making, as the threshold is lower than our cohort median (200 pg/mL) but identifies a broader population potentially benefiting from anti-cytokine therapy in line with previously reported thresholds.

**Figure 5:**
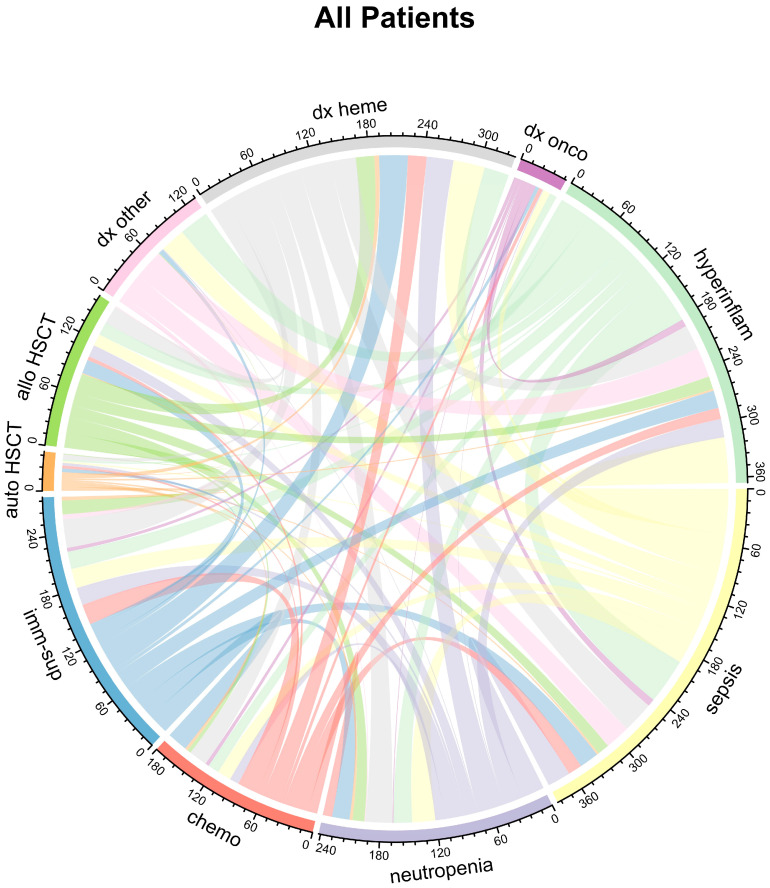

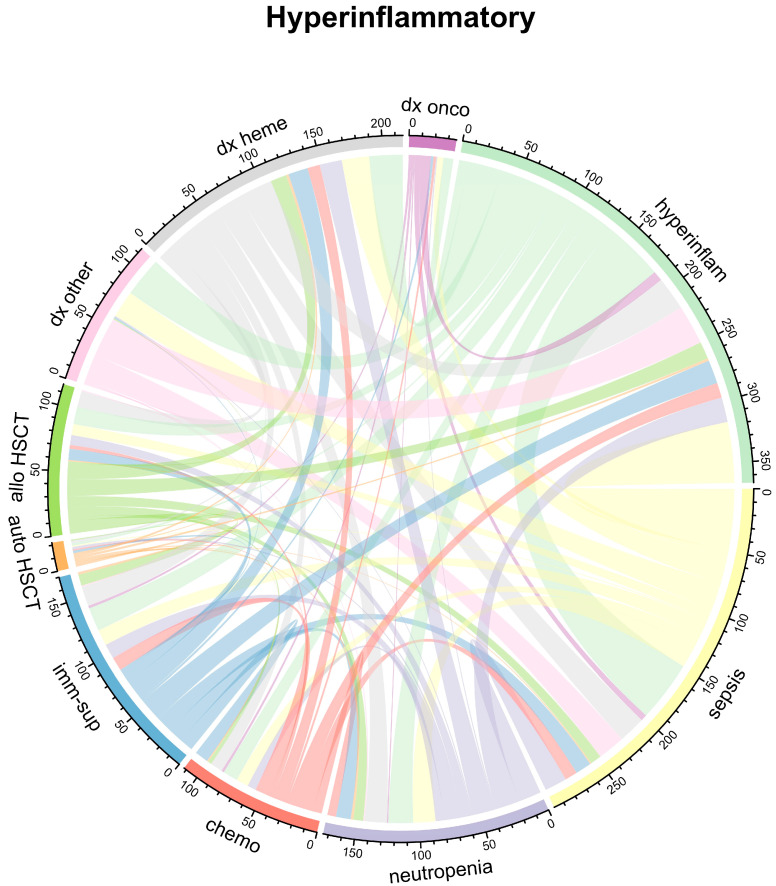

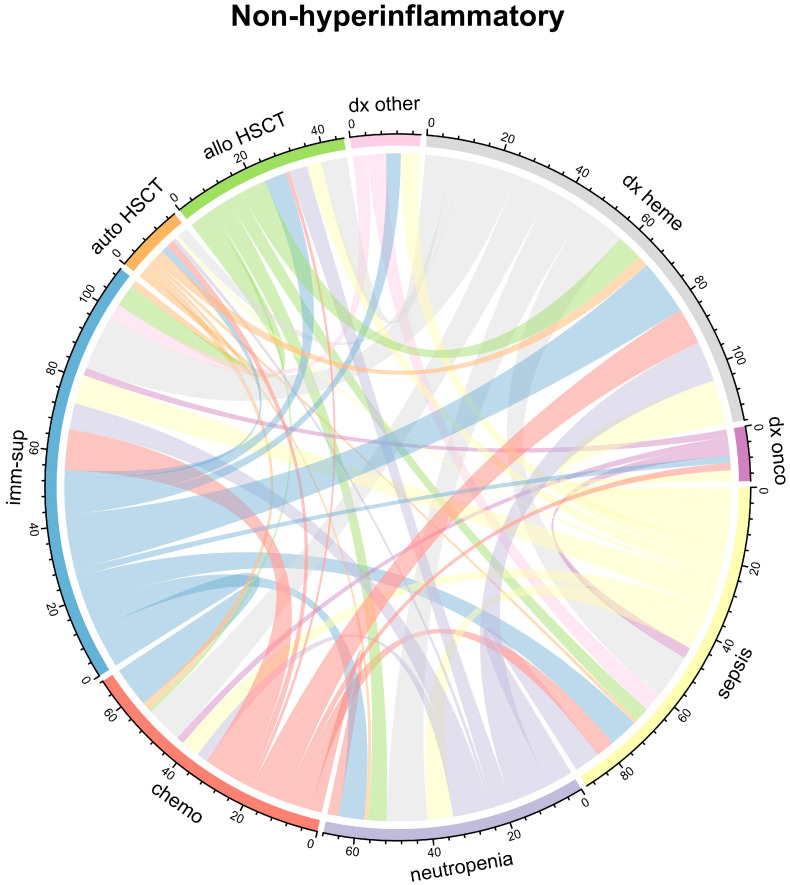
Pairwise co-occurrence of patient characteristics according to inflammatory phenotype. Left panel: all patients (*n* = 160); middle panel: patients with hyperinflammatory phenotype (IL-6 level ≥ 100 pg/mL and predicted mortality probability > 40% according to Bayesian logistic regression analysis, *n* = 67); right panel: non-hyperinflammatory patients (*n* = 93).

**Figure 6:**
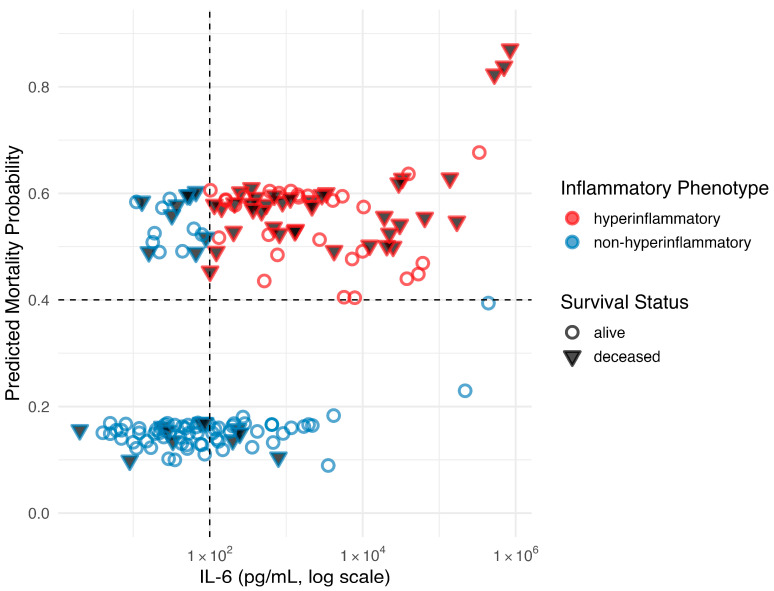
IL-6 concentrations (log scale) plotted against predicted mortality probability, colored by inflammatory phenotype, and with shapes indicating survival status. The hyperinflammatory phenotype was defined as IL-6 ≥ 100 pg/mL combined with a predicted mortality probability > 40% based on Bayesian logistic regression.

Of the 160 patients, 67 (42%) fulfilled criteria for the hyperinflammatory phenotype; this subgroup was characterized by a high incidence of sepsis, prior allogeneic HSCT, and other markers of disease severity (Table 4). In addition, patients with hyperinflammatory phenotype were more likely to require mechanical ventilation, had higher doses of vasopressors and higher levels of inflammatory markers other than IL-6, such as CRP, PCT, lactate, and cortisol (Table 4, Figure 5). Moreover, using the predicted probabilities derived from the Bayesian logistic regression and the level of IL-6 on day 1, there was a striking difference in the outcome as mortality was 58% compared to 22% in the group of hyperinflammatory compared to non-hyperinflammatory patients, respectively (*p* < 0.001, Figure 6).

### 2.4. Latent Class Analysis

To identify distinct clinical subgroups within the cohort, latent class analysis (LCA) was applied to models specifying one through six classes, with fit statistics and classification measures supporting a four-class solution as the most robust and interpretable (Table 5). Median class assignment probabilities were 100% [IQR 99.9–100%] for class 1, 100% [IQR 95.8–100%] for class 2, 100% [IQR 99.6–100%] for class 3, and 98.7% [IQR 95.9–99.9%] for class 4, with > 0.9 posterior probability observed in 80–85% of patients per class, indicating strong class separation. Sensitivity analyses, performed by iteratively removing individual indicator variables, confirmed that no single feature disproportionately influenced the model. Although sensitivity analyses suggested slight Bayesian information criterion (BIC) overparameterization, the trade-off between parsimony and interpretative depth favored clinical interpretability, reinforcing the four-class solution. Bootstrap resampling (*n* = 500) further demonstrated model stability, with the mean BIC across samples being 1590.5 (standard deviation (SD) 47.7), reflecting low relative variation (~3%). Item–response probabilities were highly stable for most variables (SD < 0.3), and median probabilities across resamples were broadly consistent with the original estimates, indicating robust and reliable class assignments (Figure S1).

**Figure 7:**
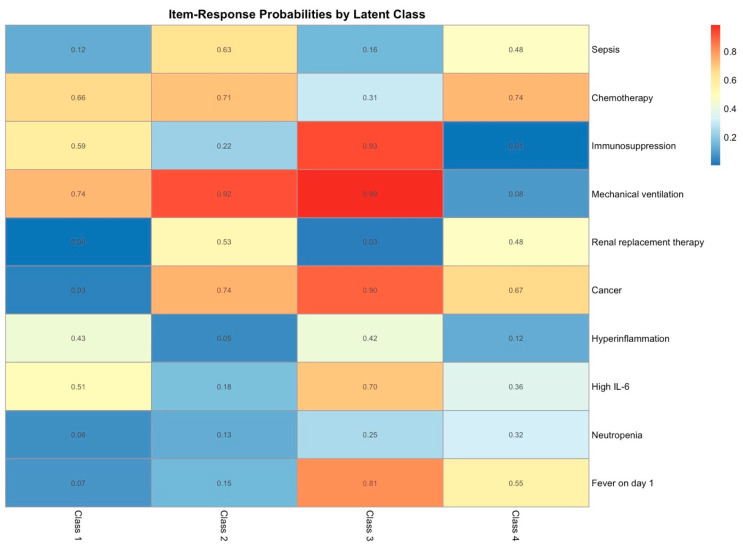
Heatmap of item–response probabilities for each latent class. Values range from 0 to 1, with higher values (yellow to red) indicating a greater probability that a patient in the given class exhibits the item, and lower values (blue) indicating a lower probability. Items include sepsis, chemotherapy, immunosuppression, mechanical ventilation, renal replacement therapy, cancer, hyperinflammation, high IL-6, neutropenia, and fever on day 1.

The heatmap of item–response probabilities (Figure 7) provides a visual summary of the defining features of each latent class, highlighting class-specific differences in clinical characteristics, which closely align with the stratified patient characteristics (Table 6). Notably, certain variables, such as immunosuppression, mechanical ventilation, and cancer, showed highly class-specific response patterns.

Overall, demographic characteristics were broadly similar across classes.

Class 1 (*n* = 66, 41.2%) comprised predominantly non-cancer patients, with no history of chemotherapy and only minimal immunosuppression (6.1%). These patients presented with low severity scores (Therapeutic Intervention Scoring System (TISS) (mean) 11.20 (SD) ± 7.76, SAPS 37.18 ± 17.45), relatively lower levels of inflammatory markers and metabolites (e.g., IL-6 day 1 274.11 ± 658.52 pg/mL), and preserved neutrophil (9.06 ± 6.09 × 10^9^/L) and leukocyte (14.77 ± 30.63 × 10^9^/L) counts without evidence of neutropenia. Fever was infrequent (12.1%), and the need for organ support was limited, as reflected by low rates of mechanical ventilation (19.7%), minimal noradrenaline requirements (2.38 ± 7.35 µg/kg/min), and shorter ICU stays (5.09 ± 6.12 days). Outcomes were favorable, with mortality at 16.7%.

Class 2 (*n* = 42, 26.2%) also consisted mainly of non-cancer patients without chemotherapy exposure or substantial immunosuppression but was distinguished by a high prevalence of sepsis (69.0%). Patients in this group exhibited the highest severity scores (TISS 20.95 ± 7.69, SAPS 58.88 ± 15.58), elevated IL-6 (11,418.14 ± 33,729.84 pg/mL) and lactate levels, and markedly increased inflammatory markers, including CRP and PCT. Neutropenia was only present in one patient (neutrophils 13.94 ± 10.36 × 10^9^/L). Mechanical ventilation was required in all patients, noradrenaline support was highest across classes (88.55 ± 153.43 µg/kg/min), and ICU stays were relatively prolonged (14.36 ± 15.82 days). This group demonstrated the poorest outcomes, with the highest mortality (59.5%).

Class 3 (*n* = 30, 18.8%) was composed exclusively of cancer patients, predominantly with hematologic malignancies, of whom nearly half had received chemotherapy and two-thirds were immunosuppressed. A substantial proportion had undergone HSCT. About half of the patients presented with sepsis. Inflammatory marker profiles were heterogeneous, with relatively moderate to high IL-6 concentrations (22,157.07 ± 88,397.60 pg/mL) but lower lactate levels and only moderately elevated CRP and PCT. Neutropenia was common (40.0%), though not universal. The need for intensive support was limited, with most patients not mechanically ventilated (73.3%) and requiring only minimal noradrenaline support. ICU stays were of moderate duration (12.13 ± 12.71 days), and mortality rates were intermediate at 40%.

Class 4 (*n* = 22, 13.8%) included exclusively cancer patients, again predominantly with hematologic malignancies. Half had received chemotherapy, two-thirds were immunosuppressed, and nearly half had undergone HSCT. The majority presented with sepsis (86.4%). Disease severity was high (TISS 19.23 ± 7.49, SAPS 63.64 ± 19.81), accompanied by the highest IL-6 concentrations (126,147.59 ± 245,161.65 pg/mL), very high CRP and PCT levels, and elevated lactate. Neutropenia was present in 81.8% of patients, with markedly reduced neutrophil and leukocyte counts. Fever was common (72.7%), and all patients required mechanical ventilation and high noradrenaline support (41.50 ± 42.89 µg/kg/min). ICU stays were the longest across classes (15.91 ± 10.57 days). Outcomes were poor, with survival and mortality both at 50% (Table 6).

## 3. Discussion

### 3.1. Study Rationale

In this study, we examined 160 critically ill patients to characterize inflammatory patterns with a particular focus on plasma IL-6, patient characteristics, and clinical outcomes. Using Bayesian logistic regression and LCA, we identified clinically distinct subgroups, including hyperinflammatory phenotypes associated with excessive mortality risk, that may represent potential targets for anti-cytokine therapy. Severe systemic inflammation is a defining feature of critical illness in the ICU, reflecting a complex host response to infectious and non-infectious stimuli. Among the cytokines orchestrating this response, IL-6 occupies a central role. IL-6 contributes to the regulation of physiological immune defense mechanisms against infection but is also implicated in maladaptive processes driving pathological hyperinflammation. Elevated IL-6 levels are a hallmark of several syndromes characterized by dysregulated immune activation, including sepsis, CRS, and HLH [7,17,18]. In sepsis, IL-6 has been studied extensively, with multiple investigations demonstrating associations between IL-6 concentrations, disease severity, and adverse outcomes [19,20,21,22]. Given these observations, IL-6 has been explored as a potential biomarker for the early diagnosis of sepsis. However, despite a large body of literature, current evidence remains inconclusive regarding its diagnostic utility [23]. Clinical relevance of IL-6 has further been underscored by its central role in CRS, particularly as a frequent and potentially life-threatening complication of CAR T-cell therapy [24]. IL-6 receptor blockade with tocilizumab, a humanized monoclonal antibody initially developed for rheumatoid arthritis, has proven highly effective in reversing this syndrome, often within a day [2]. More recently, the SARS-CoV-2 pandemic highlighted IL-6 as a key cytokine in COVID-19, where elevated IL-6 concentrations predicted the need for mechanical ventilation and were associated with hyperinflammatory states resembling CRS or HLH, raising the hypothesis that IL-6 may serve as a therapeutic target in COVID-19 analogous to its role in CRS [25,26,27,28].

### 3.2. Cohort Characteristics and Initial Inflammatory Observations

Recognizing the central role of IL-6 in systemic inflammation and the availability of targeted therapies such as tocilizumab, we sought to characterize IL-6–related inflammatory patterns in a critically ill population. Overall, the clinical characteristics of our cohort were typical for a quaternary medical ICU (MICU), although cancer patients were overrepresented (44%), given the hospital’s role as a comprehensive cancer center. IL-6 concentrations were generally high, but neither baseline IL-6 nor PCT, both often showing parallel trends, were independently associated with ICU mortality in Cox proportional hazards models or with differences in ICU survival time in Kaplan–Meier analyses. Nevertheless, when dichotomized, “high IL-6” (≥200 pg/mL) was associated with lower ICU survival in patients. Mortality overall was primarily associated with mechanical ventilation and higher SAPS II scores, consistent with established risk factors in the ICU [29,30,31,32,33,34]. As in prior reports [19,20,21], septic patients in our cohort exhibited higher IL-6 concentrations, with levels slightly higher in non-survivors compared to survivors.

### 3.3. Neutropenia as a Driver of IL-6 Dysregulation and Distinct Outcomes

IL-6 was substantially elevated in neutropenic compared to non-neutropenic patients, reaching concentrations similar to those described in severe CRS (median 8345 pg/mL, range 1088–37,324) [35]. Lasso regression confirmed neutrophil count as a major determinant of IL-6 levels. This finding implies that neutropenic patients display a more intense systemic inflammatory response than non-neutropenic patients, which may in part reflect differences in sepsis frequency and disease severity, as SAPS II scores were significantly higher in neutropenic individuals. In line with this, other inflammatory markers, including CRP and PCT, were also higher in neutropenic patients. We further observed a consistent relationship between IL-6 and PCT across most patients; however, in neutropenic individuals, IL-6 rose disproportionately compared to PCT, suggesting differential regulation of the inflammatory response, and neutropenia was independently associated with IL-6 levels in multivariate analysis. These observations align with prior work by Reilly et al., who also reported disproportionately elevated IL-6 in neutropenic individuals, and support the concept that neutropenic sepsis is biologically and clinically distinct from sepsis in non-neutropenic patients [36]. Indeed, IL-6 levels were highest in neutropenic patients with concomitant sepsis, far exceeding levels observed in patients with either neutropenia or sepsis alone. The mechanisms underlying this observation remain incompletely understood. Because most cases of neutropenia in this cohort were chemotherapy-induced, the relevant source of IL-6 could be neutrophils themselves or other immune cells also affected by chemotherapy. IL-6 can be produced by several blood cell types, including T and B lymphocytes and monocytes. Neutrophils, however, have also been shown to down-regulate IL-6 production [37]. Thus, the absence of neutrophils may contribute to unchecked IL-6 release, potentially as part of a compensatory feedback loop aimed at stimulating neutrophil production and recruitment to sites of inflammation [38]. Notably, chemotherapy per se was not associated with higher IL-6 concentrations in our cohort, indicating that these elevations are unlikely to reflect direct effects of chemotherapy reported elsewhere [39,40,41]. Importantly, while neutropenia was associated with highly elevated IL-6, this did not translate into significant differences in IL-6 concentrations between survivors and non-survivors within the neutropenic subgroup. In fact, Bayesian logistic regression even suggested a possible, though uncertain, protective association of neutropenia with mortality (OR = 0.74). This counterintuitive protective association of neutropenia likely reflects a combination of confounding factors, including the younger age of hematologic patients (median age 50 vs. 61 years in non-neutropenic patients), different goals of care and treatment intensity in cancer patients, selection bias toward patients stable enough for aggressive care, and potential survival bias. Future analyses should stratify by age, malignancy type, and treatment goals to isolate the true effect of neutropenia on outcomes.

### 3.4. IL-6 Prognostic Limitations and the Need for Multi-Cytokine Approaches

While IL-6 is a central mediator of hyperinflammatory responses and a well-established marker in syndromes such as sepsis, CRS, and HLH [7,17,18], growing evidence indicates that its prognostic capacity is enhanced when analyzed within a multi-cytokine context rather than in isolation [42]. Complex cytokine ratios have demonstrated superior predictive accuracy compared to traditional severity scores (APACHE II, SAPS II, and SOFA), individual cytokines, and simple inflammatory markers [43]. Although IL-6 remains a key inflammatory mediator, its prognostic accuracy has been shown to vary considerably across studies and patient cohorts [44,45]. Even where IL-6 outperforms classic inflammatory markers such as CRP, its performance metrics typically reach only moderate levels, emphasizing the need to consider interactions between complementary inflammatory mediators [46]. Recent investigations in sepsis, ARDS, and COVID-19 have consistently shown that integrating IL-6 with cytokines, such as IL-8, TNF-α, and IL-10, enhances discrimination for mortality, organ failure, and disease severity [47,48,49,50,51]. Imbalances of IL-10, CCL2, CXCL9, CXCL10, and IL-1β have also been strongly associated with adverse outcomes among critically ill patients, reflecting the multifaceted nature of immune dysregulation [52,53]. In particular, ratios such as IL-6/IL-10 and TNF-α/IL-10 more effectively capture the dynamic balance between pro- and anti-inflammatory pathways than absolute cytokine concentrations alone [54,55,56]. Likewise, immune cell-normalized indices, including IL-6/lymphocyte or IL-10/lymphocyte ratios, have emerged as reliable predictors of ICU mortality, further supporting composite approaches [54,57,58]. Beyond ratio-based metrics, broader cytokine panels and multi-analyte signatures appear to yield even greater prognostic resolution. Studies incorporating multiple cytokines, such as IL-6, IL-10, IFN-γ, TNF-α and IL-17A have linked persistent co-elevations to organ injury and disease severity [48,49]. These multi-marker patterns likely reflect underlying immune network activation more accurately than any single cytokine measurement can achieve. Computational approaches, including machine learning-driven models, now enable identification of such high-dimensional biomarker interactions with promising results for clinical translation [59,60,61].

In light of these advances, we believe that integrating IL-6 with additional inflammatory cytokines or chemokines could refine the prognostic insights derived from our study. Multi-marker strategies may help delineate patient subgroups with distinct inflammatory trajectories, enabling more personalized risk stratification and therapeutic targeting. While our present analysis focused on IL-6 as an individual marker due to data availability, future work should extend toward comprehensive cytokine profiling—integrating IL-6 with complementary mediators such as IL-8, IL-10, or TNF-α—to establish more reliable prognostic signatures in critical illness. This multi-dimensional framework, ideally combined with computational modeling, could reveal immunological patterns that single biomarkers fail to capture and ultimately improve clinical prediction and intervention strategies.

### 3.5. Uncovering Hyperinflammatory Phenotypes and Clinical Subgroups

Moving beyond single biomarkers, we sought to identify clinically meaningful inflammatory subgroups. We defined two broad phenotypes: a hyperinflammatory phenotype (IL-6 ≥ 100 pg/mL, predicted mortality probability > 40%) characterized by sepsis, prior allogeneic HSCT, mechanical ventilation, vasopressor requirement, and elevated inflammatory markers other than IL-6 (CRP, PCT, lactate, and cortisol), and a non-hyperinflammatory phenotype with lower disease burden. Outcomes differed markedly: mortality was 58% in the hyperinflammatory phenotype compared to 22% in the non-hyperinflammatory phenotype.

This dichotomy aligns with a broader literature demonstrating that circulating protein signatures, whether derived from untargeted proteomics [62] or targeted panels of cytokines and inflammatory mediators, similarly resolve into reproducible inflammatory subgroups. Most analyses converge on two or three distinct profiles, with those exhibiting the highest circulating inflammatory mediators representing patients at greatest risk of poor outcomes [63,64,65]. The clearest framework for such stratification comes from studies in ARDS, where combining biomarker panels with clinical variables has led to the widely recognized hyper- and hypoinflammatory phenotypes [66,67,68,69,70,71,72]. Notably, these groups are not limited to ARDS: given the biological and clinical overlap between ARDS and sepsis, parallel inflammatory patterns have also been demonstrated in septic cohorts [65]. The hyperinflammatory profile is typically marked by elevated IL-6, IL-8, and soluble TNF receptor-1, accompanied by reductions in protein C and bicarbonate [4].

In extending these insights, we refined the hyperinflammatory pattern by identifying subgroups potentially amenable to targeted anti-cytokine strategies. Using LCA, we were able to distinguish clinically distinct subgroups within our cohort, ranging from relatively stable patients with low inflammatory activity and high survival, to severely inflamed groups with markedly increased mortality. Broadly, classes 1 and 2 were composed mainly of non-cancer patients without neutropenia, whereas classes 3 and 4 consisted almost exclusively of cancer patients, many of whom were neutropenic, likely reflecting underlying disease and treatment effects [73]. Within these strata, further distinctions emerged: classes 1 and 3 exhibited lower disease burden, while classes 2 and 4 were characterized by higher disease burden and greater sepsis prevalence. In simplified terms, this yielded four clinically distinct combinations: (1) no neutropenia/no sepsis/low disease burden, (2) no neutropenia/sepsis/high disease burden, (3) neutropenia/infrequent sepsis/low disease burden, and (4) neutropenia/sepsis/high disease burden. Mapping these findings onto our Bayesian models, the previously defined hyperinflammatory phenotype most likely corresponded primarily to classes 2 and 4. LCA further resolved this phenotype into two subtypes: an “IL-6 moderate” group with sepsis but no neutropenia (class 2), and an “IL-6 high” group with both sepsis and neutropenia (class 4). As expected, IL-6 levels rose in sepsis and increased further when neutropenia was present. Despite these differences in IL-6 magnitude, mortality was similar across both classes, underscoring the limited prognostic value of IL-6 alone. This modest prognostic utility as an isolated biomarker becomes particularly apparent when contrasted with clinical parameters: mechanical ventilation emerged as the strongest predictor of mortality (Bayesian OR 7.25, Cox PH OR 8.78), substantially exceeding IL-6’s predictive capacity (Bayesian log-IL-6 OR 1.07). These results reinforce the importance of contextualizing inflammatory biomarkers such as IL-6 within accompanying clinical parameters, particularly organ dysfunction requiring ventilatory support, to accurately stratify risk and guide therapeutic decisions. Interestingly, while dichotomized IL-6 retains a modest association with mortality, neutropenia, though useful for subdividing the hyperinflammatory phenotype, did not meaningfully affect outcomes (*p* = 0.5).

For pragmatic application, a simplified bedside scoring system could incorporate readily available parameters: mechanical ventilation (2 points), SAPS II > 50 (1 point), sepsis (1 point), and neutropenia (1 point). Patients scoring ≥ 3 points with IL-6 ≥ 100 pg/mL would qualify for the hyperinflammatory phenotype, pending validation in prospective cohorts.

From an implementation standpoint, real-time implementation necessitates point-of-care or rapid IL-6 testing (2–4 h turnaround), integrated with extant severity scoring, clinical decision support frameworks, and standardized anti-IL-6 therapy protocols. Feasibility in practice is ultimately contingent on laboratory resources and must be confirmed across settings with varying infrastructure capabilities.

### 3.6. Translational Implications for Targeted Anti-Cytokine Therapy

Clinically, these findings have notable translational implications. Multiple studies in ARDS and sepsis have shown that hyper- and hypoinflammatory phenotypes not only differ in outcomes but also in their response to therapy, including fluid resuscitation, statins, corticosteroids, and activated protein C [65,69,71,72,74]. IL-6 receptor antagonists have already demonstrated clinical benefit in critically ill patients with COVID-19, improving organ support-free days, reducing progression to invasive ventilation, and lowering mortality [75,76]. Our data indicate that septic patients with neutropenia, who display exceptionally high IL-6 levels and poor outcomes, may derive the greatest benefit from such targeted therapy. Therefore, anti-IL-6 therapy may be considered for critically ill patients who meet all of the following criteria: IL-6 levels ≥ 100 pg/mL, predicted mortality > 40%, concurrent sepsis, and neutropenia. Clinical decisions should further factor in IL-6 testing turnaround time, the patient’s clinical trajectory, and contraindications. Early identification, ideally within 48 h of ICU admission, appears most promising, as supported by recent tocilizumab trials in COVID-19 [77].

In parallel to cytokine-based stratification, single-cell transcriptomic profiling of platelets in COVID-19, sepsis, and systemic lupus erythematosus (SLE) has resolved discrete “fatal” and “survival” platelet subpopulations defined by 21 consensus biomarkers using Deep Neural Network (DNN) and eXtreme Gradient Boosting (XGB) machine learning models. Fatal clusters overexpress hypoxia-responsive, coagulation, and endothelial-adhesion gene modules, whereas survival clusters are enriched for cytoplasmic translation and adaptive-immune programs. The platelet-to-T cell ratio further stratifies mortality risk independently of IL-6 levels [78]. IL-6 is now increasingly recognized as a pleiotropic immunothrombotic cytokine that intricately interacts with platelets by promoting thrombopoiesis and lowering the threshold for platelet activation, thus priming platelets for hyper-responsiveness and accelerating thrombus formation. Pharmacological inhibition of IL-6 signaling, such as with tocilizumab, continues to be investigated specifically for its potential to mitigate thrombotic complications in severe inflammatory states. These IL-6-mediated mechanisms of platelet hyperactivity likely contribute to the disproportionate IL-6 elevations observed in our neutropenic and septic subgroups, reflecting a bidirectional relationship whereby platelets both respond to and modulate IL-6-mediated inflammation through direct interactions with endothelial and immune cells [78,79,80]. Integrating platelet transcriptomic signatures with our Bayesian and LCA-derived IL-6 phenotypes could thus enhance early risk stratification and improve prognostic accuracy. Moreover, these findings suggest that combined anti-IL-6 therapy and targeted modulation of fatal platelet states, such as heparanase inhibition or actin-cytoskeleton remodelers, may synergistically attenuate immunothrombosis in hyperinflammatory patients (IL-6 ≥ 100 pg/mL with elevated fatal-platelet biomarkers).

### 3.7. Limitations, Unresolved Mechanisms, and Future Validation Needs

Taken together, moving beyond arbitrary cutoffs or simple dichotomization allows for the definition of clinically meaningful inflammatory subphenotypes. Such stratification could help guide anti-cytokine therapies, as different subphenotypes may vary in their inflammatory profiles and treatment responses. More broadly, these findings highlight the potential of precision medicine in critical illness, where tailoring interventions to underlying inflammatory phenotypes could improve patient outcomes. Future research should seek to clarify the mechanistic relationship between neutropenia and IL-6 dysregulation—specifically, whether IL-6 elevation represents a compensatory response to neutrophil depletion or underlying disease pathophysiology, the contribution of non-neutrophil immune cells to IL-6 production during neutropenia, and the longitudinal dynamics of IL-6 as neutrophil counts recover.

This study has several limitations. It was a single-center, retrospective analysis with a moderate sample size, limiting statistical power to detect smaller effects. Only short-term outcomes were assessed, and generalizability is naturally restricted by the high proportion of cancer patients. Moreover, in the studied cohort, mechanical ventilation was both highly prevalent within the hyperinflammatory phenotype and strongly weighted in the mortality prediction model, raising the potential for circularity in phenotype characterization. Although mechanical ventilation is one of the most powerful individual predictors of mortality, it is not alone sufficient to define either the phenotype or the outcome. The predictive value observed derives from the integrated model incorporating all available variables, which demonstrated superior performance in sensitivity analyses; nonetheless, the strong influence of mechanical ventilation warrants cautious interpretation of these findings.

To advance clinical translation and broader applicability, future multicenter validation efforts should prioritize several key elements. First, standardization of IL-6 measurement platforms across participating centers is essential to address inter-platform variability and ensure comparable results, as differences in assay methodologies may significantly affect the clinical applicability of the established IL-6 threshold. Second, validation in diverse ICU populations beyond quaternary cancer centers will be necessary to assess generalizability, given that our cohort was enriched for cancer patients (44% overall) and may not reflect the typical case mix of general medical ICUs. Third, prospective validation of the hyperinflammatory phenotype definition is required to confirm its utility in real-time clinical decision-making, as our retrospective analysis may be subject to classification bias. Finally, assessment of the herein established IL-6 threshold (≥100 pg/mL) across different healthcare systems with varying IL-6 testing capabilities and turnaround times will be crucial for determining the practical feasibility of phenotype-guided therapy in diverse clinical settings.

## 4. Materials and Methods

### 4.1. Patient Population and Study Design

This is a retrospective analysis including data of consecutive patients treated at a MICU of an academic quaternary medical center with a comprehensive cancer program (University Hospital Cologne, Cologne, Germany).

The study was approved by the institutional ethics committee (#19-1155), and due to the retrospective nature of the analysis, the requirement for individual informed consent was waived.

Data were collected by reviewing the medical records and extracting baseline patient characteristics, including age, sex, type of underlying disease, length of ICU stay, interventions in the ICU (vasopressor use, mechanical ventilation, and renal replacement therapy), ICU survival, chemotherapeutic treatment, TISS, and SAPS. The following laboratory values on the day of ICU admission and following 24 h after admission were included: IL-6 level, lactate, cortisol, CRP, PCT, leukocyte count, and neutrophil count. Neutropenia was defined as less than 1.0 neutrophils ×10^9^/L. Patients on chemotherapy were defined as those patients who had received antineoplastic treatment within the last 30 days prior to ICU admission. Patients who received antibiotics (excluding prophylactic treatment) and underwent sampling of body fluids for culturing were defined as patients with suspected infection, and patients with “life-threatening organ dysfunction caused by a dysregulated host response to infection” were defined as patients with sepsis according to current Surviving Sepsis Campaign guidelines and recommendations [81,82]. Immunosuppression was defined as medication with immunosuppressive drugs such as calcineurin inhibitors as cyclosporine, m-TOR inhibitors as tacrolimus, or corticosteroids (≥20 mg prednisolone/d equivalent ≥ 14 days), and/or in case of neutropenia.

### 4.2. IL-6 Measurement and Quality Control

Plasma IL-6 concentrations were measured using an electrochemiluminescence immunoassay (sandwich immunoassay with electrochemiluminescence detection, ECLIA) on the Cobas E801 platform by Roche Diagnostics. The assay is calibrated according to the international World Health Organization (WHO) standard NIBSC 1st IS 89/548 and accredited according to the German DIN ISO EN 15189 norm.

### 4.3. Statistical Analysis

Patient characteristics are reported as the number [percentage] for categorical variables and as mean or median [SD or IQR] for continuous variables. Comparisons between patient subsets were performed using Pearson’s chi-square test or Fisher’s exact test for categorical variables and analysis of variance (ANOVA), the Kruskal–Wallis test, or the Wilcoxon rank-sum test for continuous variables. A *p* value < 0.05 was considered statistically significant.

Patients were categorized based on plasma IL-6 levels measured on day 1 of ICU admission as either “IL-6 high” (≥200 pg/mL) or “IL-6 low” (<200 pg/mL). The cut-off approximates the cohort median and aligns with common literature values around 200 pg/mL [83,84,85,86].

Multivariable adaptive Lasso regression was performed using log-transformed IL-6 as the outcome and relevant clinical and laboratory variables as predictors. Variables included in the multivariable analyses were checked for collinearity and not included in the multivariable model in case of collinearity. Variables selected by the adaptive Lasso were included in a linear regression model to estimate coefficients, 95% CI, and *p* values. Results are presented as exponentiated coefficients (Exp(Beta)), representing multiplicative effects on IL-6 levels.

Kaplan–Meier survival analyses and multivariate Cox proportional hazards regression analyses were conducted to investigate factors associated with time to death.

Bayesian logistic regression analysis was applied to identify patients with high mortality and distinct laboratory and clinical features, implying a hyperinflammatory phenotype. The association between clinical factors and in-hospital mortality was analyzed by using a Bayesian logistic regression estimating posterior medians and 95% credible intervals, with weakly informative priors (Normal (0, 2.5) for regression coefficients). Candidate variables included all available demographic, diagnostic, and laboratory variables as predictors of mortality as the primary outcome. The final Bayesian model was selected by including all candidate variables in the model and stepwise exclusion of variables and applying posterior predictive checks for model calibration and to assess fit. In addition, stepwise logistic regression with a generalized linear model (glm) and random forest analysis were applied to check for the plausibility of variables associated with mortality in the final model. Finally, sensitivity analysis was performed by testing the inclusion of all variables as predictors and alternative priors, and different mortality thresholds. Patients were defined as having a hyperinflammatory phenotype, in case of Bayesian predicted mortality probability > 40% and plasma IL-6 level ≥ 100 pg/mL on day 1 of ICU admission.

To identify distinct clinical subgroups within the cohort, LCA [87] was performed using ten dichotomized indicators that have been implicated as predictive features: sepsis, cancer, chemotherapy, mechanical ventilation, renal replacement therapy, hyperinflammation, elevated IL-6, immunosuppression, neutropenia, and fever on day 1. Models specifying one to six latent classes were evaluated, with selection guided by fit indices including the Akaike information criterion (AIC) [88], sample size-adjusted Bayesian information criterion (aBIC) [89,90], likelihood ratio/deviance statistic (G^2^), *p* values from likelihood ratio tests comparing k versus k–1 classes, and entropy [91] to assess classification certainty. The final number of classes was determined based on a balance of statistical fit, parsimony, and clinical interpretability. Posterior probabilities of class membership were calculated for each patient, and median assignment probabilities were used to evaluate classification quality. Sensitivity analyses, performed by iteratively removing individual indicators, assessed the influence of each variable on the model. Model stability was further examined using 500 bootstrap resamples, from which median, mean, SD, and 95% CI of item–response probabilities were derived. Heatmaps were generated to visualize class-specific item–response probabilities and to illustrate the robustness of assignments across bootstrap samples.

All statistical analyses of the data were performed using R version 4.3.1 and 4.4.1 [92].

## 5. Conclusions

In this ICU cohort, IL-6 concentrations were markedly elevated in patients with sepsis and in those with neutropenia, with the highest levels observed in individuals affected by both. Despite these pronounced elevations, IL-6 alone provided only limited prognostic information for mortality, and neutropenia, while strongly influencing IL-6 levels, did not independently affect outcomes. LCA revealed clinically meaningful inflammatory subgroups, ranging from patients with relatively low inflammatory activity and favorable survival to those with severe hyperinflammation and markedly increased mortality. Hyperinflammatory subtypes were strongly linked to sepsis and neutropenia, yet mortality risk was largely determined by other clinical factors, particularly the need for mechanical ventilation. By extending the analysis of IL-6-driven inflammatory patterns to a specialized ICU population with a high prevalence of malignancy, our study underscores the complex and multifactorial nature of systemic inflammation and highlights the potential for phenotype-guided approaches to refine risk stratification and guide precision therapy in critically ill patients.

## Figures and Tables

**Table 1:** Patient characteristics stratified by level of plasma IL-6 on day 1 of ICU (IL-6 high = IL-6 ≥ 200 pg/mL, IL-6 low = IL-6 < 200 pg/mL).

Characteristic	Overall ^1^ *n* = 160	IL-6 High ^1^ *n* = 80	IL-6 Low ^1^ *n* = 80	*p* Value ^2^
Sex: female	52 (33%)	25 (31%)	27 (34%)	0.7
Age [years]	59 [44, 69]	60 [44, 69]	58 [45, 70]	0.8
Diagnosis				0.5
Other	89 (56%)	41 (51%)	48 (60%)	
Hematologic	53 (33%)	30 (38%)	23 (29%)	
Oncologic	18 (11%)	9 (11%)	9 (11%)	
Chemotherapy	24 (15%)	14 (18%)	10 (13%)	0.4
Immunosuppression	40 (25%)	20 (25%)	20 (25%)	>0.9
Autologous HSCT	5 (3.1%)	2 (2.5%)	3 (3.8%)	>0.9
Allogeneic HSCT	21 (13%)	13 (16%)	8 (10%)	0.2
Sepsis	69 (43%)	50 (63%)	19 (24%)	<0.001
Mechanical ventilation	85 (53%)	59 (74%)	26 (33%)	<0.001
Renal replacement therapy	18 (11%)	13 (16%)	5 (6.3%)	0.045
Noradrenaline day 1 [µg /kg/min]	0 [0, 16]	10 [0, 66]	0 [0, 1]	<0.001
TISS	15 [9, 19]	18 [11, 25]	10 [5, 15]	<0.001
SAPS	46 [33, 64]	59 [43, 71]	34 [27, 53]	<0.001
Fever on day 1	44 (28%)	35 (44%)	9 (11%)	<0.001
IL-6 [pg/mL]	200 [42, 1576]	1576 [569, 15,748]	42 [19, 83]	<0.001
Lactate [mmol/L]	2.1 [1.3, 3.9]	2.4 [1.4, 5.8]	1.8 [1.2, 2.8]	0.011
CRP [mg/L]	105 [27, 220]	158 [87, 303]	41 [11, 139]	<0.001
PCT [ng/mL]	1 [0, 16]	9 [1, 43]	0 [0, 1]	<0.001
Cortisol [nmol/L]	328 [200, 513]	436 [307, 651]	232 [130, 391]	<0.001
Neutrophils [×10^9^/L]	6 [1, 12]	6 [0, 16]	7 [2, 12]	0.6
Neutropenia [<1.0 × 10^9^/L]	31 (19%)	23 (29%)	8 (10%)	0.003
Leukocytes [×10^9^/L]	9 [3, 17]	7 [1, 18]	9 [5, 16]	0.083
ICU LOS [days]	5 [3, 14]	8 [3, 17]	4 [3, 9]	0.007
Survival	101 (63%)	41 (51%)	60 (75%)	0.002

^1^ *n* (%); median [IQR]. ^2^ Pearson’s chi-squared test; Wilcoxon rank-sum test; Fisher’s exact test. HSCT hematopoietic stem cell transplantation, TISS Therapeutic Intervention Scoring System, SAPS Simplified Acute Physiology Score II, IL-6 interleukin-6, CRP C-reactive protein, PCT procalcitonin, ICU intensive care unit, LOS length of stay.

**Table 2:** Adaptive Lasso linear regression applied to log-transformed IL-6 levels on day 1 (pg/mL, only significant variables shown). Assuming all other predictors are held constant, Beta represents the estimated change in the log of IL-6 concentration (log[IL-6]) resulting from a one-unit increase in the corresponding predictor variable, and Exp(beta) represents the multiplicative (fold) change in the expected IL-6 concentration associated with a one-unit increase in the predictor.

Characteristic	Beta	95% CI	*p* Value	Exp (Beta)	Exp (95% CI)
Mechanical ventilation	0.80	0.02, 1.6	0.046	2.22	1.02–4.88
SAPS	0.04	0.02, 0.06	<0.001	1.04	1.02–1.06
Lactate [mmol/L]	0.16	0.08, 0.24	<0.001	1.18	1.08–1.28
CRP [mg/L]	0.01	0.00, 0.01	<0.001	1.01	1–1.01
Neutrophils [×10^9^/L]	−0.05	−0.09, 0.00	0.045	0.95	0.91–1

CI confidence interval, SAPS Simplified Acute Physiology Score II, CRP C-reactive protein.

**Table 3:** Posterior summaries of Bayesian logistic regression estimating the probability of mortality from six clinical variables. Parameter indicates the predictor variable. log(OR) Mean/log(OR) Median: posterior mean and median estimates of the log odds ratio (log(OR)) for each parameter. OR Mean: exponentiated mean of the log odds ratio; represents the average posterior odds ratio estimate. OR 2.5% and OR 97.5%: lower and upper bounds of the 95% credible interval for the odds ratio (obtained by exponentiating the 2.5th and 97.5th posterior quantiles).

Parameter	log(OR) Mean	log(OR) Median	OR Mean	OR 2.5%	OR 97.5%
Sex: male	0.255	0.255	1.291	0.557	2.873
Age [years]	0.002	0.002	1.002	0.980	1.025
(log) IL-6	0.067	0.067	1.070	0.909	1.261
Neutropenia	−0.298	−0.302	0.742	0.262	2.143
Fever on day 1	−0.033	−0.034	0.967	0.407	2.289
Mechanical ventilation	1.980	1.968	7.246	3.140	17.000

IL-6 interleukin-6.

**Table 4:** Patient characteristics stratified by inflammatory phenotype (only significant differences shown). The hyperinflammatory phenotype was defined by an IL-6 concentration ≥ 100 pg/mL combined with a predicted mortality probability > 40%, as estimated by Bayesian logistic regression.

Characteristic	Overall ^1^ *n* = 160	Hyperinflammatory Phenotype ^1^ *n* = 67	Non-Hyperinflammatory Phenotype ^1^ *n* = 93	*p* Value ^2^
Sepsis	69 (43%)	49 (73%)	20 (22%)	<0.001
Mechanical ventilation	85 (53%)	67 (100%)	18 (19%)	<0.001
Noradrenaline day 1 [µg/kg/min]	0 [0, 16]	17 [6, 97]	0 [0, 0]	<0.001
TISS	15 [9, 19]	19 [15, 26]	10 [5, 15]	<0.001
SAPS	46 [33, 64]	61 [46, 71]	35 [28, 52]	<0.001
Fever on day 1	44 (28%)	32 (48%)	12 (13%)	<0.001
IL-6 [pg/mL]	200 [42, 1576]	1452 [400, 20,659]	51 [22, 148]	<0.001
Lactate day 1 [mmol/L]	2.1 [1.3, 3.9]	2.5 [1.5, 6.4]	1.7 [1.1, 2.6]	<0.001
CRP day 1 [mg/L]	105 [27, 220]	172 [79, 303]	64 [13, 143]	<0.001
PCT day 1 [ng/mL]	1 [0, 16]	11 [1, 45]	0 [0, 3]	<0.001
Cortisol day 1 [nmol/L]	328 [200, 513]	485 [322, 710]	248 [142, 402]	<0.001
Allogeneic HSCT	21 (13%)	14 (21%)	7 (7.5%)	0.013
ICU LOS [days]	5 [3, 14]	12 [6, 21]	3 [3, 6]	<0.001
Mortality	59 (37%)	39 (58%)	20 (22%)	<0.001
Predicted Mortality Probability [%]	48 [15, 57]	57 [54, 61]	16 [14, 19]	<0.001

^1^ *n* (%); median [IQR]. ^2^ Pearson’s chi-squared test; Wilcoxon rank-sum test. TISS therapeutic Intervention Scoring System, SAPS Simplified Acute Physiology Score II, IL-6 interleukin-6, CRP C-reactive protein, PCT procalcitonin, HSCT hematopoietic stem cell transplantation, ICU intensive care unit, LOS length of stay.

**Table 5:** Latent class analysis (LCA) model fit statistics for models specifying one through six classes. Shown are the number of patients per class, number of estimated parameters (npar), Akaike information criterion (AIC), sample size-adjusted Bayesian information criterion (aBIC), likelihood ratio/deviance statistic (G^2^), *p* value from the likelihood ratio test (LRT) comparing k versus k–1 classes, and entropy (0–1), which reflects classification certainty.

No. of Latent Classes (k)	No. of Patients per Class	npar	AIC	aBIC	G^2^	*p* Value LRT	Entropy
1	160	10	1892.395	1891.490	657.90	-	-
2	67, 93	21	1625.818	1623.919	369.32	<0.001	1.00
3	74, 38, 48	32	1550.623	1547.729	272.13	<0.001	0.95
4	30, 66, 22, 42	43	1495.672	1491.782	195.17	<0.001	0.94
5	19, 69, 22, 9, 41	54	1498.887	1494.003	176.40	0.07	0.96
6	38, 23, 12, 9, 20, 58	65	1509.001	1503.122	164.50	0.372	0.97

**Table 6:** Differences in clinical and laboratory characteristics between latent subclasses among ICU patients. Classes were derived from a 4-class latent class analysis (LCA).

Characteristic	Class 1 ^1^ *n* = 66	Class 2 ^1^ *n* = 42	Class 3 ^1^ *n* = 30	Class 4 ^1^ *n* = 22	*p* Value ^2^
Sex					0.466
Female	22 (33.3)	11 (26.2)	9 (30.0)	10 (45.5)	
Male	44 (66.7)	31 (73.8)	21 (70.0)	12 (54.5)	
Age [years]	56.35 (18.01)	58.69 (14.54)	55.70 (17.66)	48.55 (16.81)	0.154
Diagnosis					<0.001
Other	57 (86.4)	32 (76.2)	0 (0.0)	0 (0.0)	
Hematologic	4 (6.1)	4 (9.5)	25 (83.3)	20 (90.9)	
Oncologic	5 (7.6)	6 (14.3)	5 (16.7)	2 (9.1)	
Chemotherapy	0 (0.0)	0 (0.0)	13 (43.3)	11 (50.0)	<0.001
Immunosuppression	4 (6.1)	1 (2.4)	20 (66.7)	15 (68.2)	<0.001
Autologous HSCT	1 (1.5)	0 (0.0)	3 (10.0)	1 (4.5)	0.080
Allogeneic HSCT	0 (0.0)	3 (7.1)	10 (33.3)	8 (36.4)	<0.001
Sepsis	5 (7.6)	29 (69.0)	16 (53.3)	19 (86.4)	<0.001
Mechanical ventilation	13 (19.7)	42 (100.0)	8 (26.7)	22 (100.0)	<0.001
Renal replacement therapy	3 (4.5)	8 (19.0)	3 (10.0)	4 (18.2)	0.084
Noradrenaline day 1 [µg/kg/min]	2.38 (7.35)	88.55 (153.43)	3.13 (7.50)	41.50 (42.89)	<0.001
Fever day 1	8 (12.1)	16 (38.1)	4 (13.3)	16 (72.7)	<0.001
TISS	11.20 (7.76)	20.95 (7.69)	12.00 (9.86)	19.23 (7.49)	<0.001
SAPS	37.18 (17.45)	58.88 (15.58)	46.57 (21.26)	63.64 (19.81)	<0.001
IL-6 day 1 [pg/mL]	274.11 (658.52)	11,418.14 (33,729.84)	22,157.07 (88,397.60)	126,147.59 (245,161.65)	<0.001
IL-6 level					<0.001
High	15 (22.7)	37 (88.1)	6 (20.0)	22 (100.0)	
Low	51 (77.3)	5 (11.9)	24 (80.0)	0 (0.0)	
Lactate day 1 [mmol/L]	2.45 (2.76)	5.34 (5.14)	2.59 (2.23)	4.72 (6.31)	0.001
Lactate day 2 [mmol/L]	2.15 (2.22)	4.80 (3.79)	2.62 (2.79)	4.31 (5.09)	<0.001
CRP day 1 [mg/L]	78.10 (82.51)	163.56 (125.65)	124.32 (102.37)	257.41 (127.23)	<0.001
CRP day 2 [mg/L]	88.46 (84.73)	191.00 (110.94)	134.55 (105.46)	282.04 (115.57)	<0.001
PCT day 1 [ng/mL]	3.74 (10.30)	42.00 (75.62)	23.25 (60.50)	41.72 (57.58)	0.001
PCT day 2 [ng/mL]	5.57 (12.31)	40.93 (57.93)	41.94 (99.63)	91.31 (101.79)	<0.001
Cortisol day 1 [nmol/L]	316.96 (368.28)	558.39 (450.90)	337.91 (278.34)	736.87 (609.39)	<0.001
Neutrophils [×10^9^/L]	9.06 (6.09)	13.94 (10.36)	5.02 (7.22)	0.68 (1.65)	<0.001
Neutropenia	0 (0.0)	1 (2.4)	12 (40.0)	18 (81.8)	<0.001
Leukocytes [×10^9^/L]	14.77 (30.63)	17.92 (13.57)	15.38 (30.12)	3.84 (13.63)	0.196
ICU LOS [days]	5.09 (6.12)	14.36 (15.82)	12.13 (12.71)	15.91 (10.57)	<0.001
Mortality	11 (16.7)	25 (59.5)	12 (40)	11 (50)	<0.001

^1^ mean (SD); *n* (%). ^2^ chi-squared test; Fisher’s exact test; ANOVA; Kruskal–Wallis test. HSCT hematopoietic stem cell transplantation, IL-6 interleukin-6, CRP C-reactive protein, PCT procalcitonin, TISS therapeutic Intervention Scoring System, SAPS Simplified Acute Physiology Score II, ICU intensive care unit, LOS length of stay.

## Data Availability

The datasets used and/or analyzed during the current study are available from the corresponding author on reasonable request.

## Supplementary Materials

The following supporting information can be downloaded at: https://www.mdpi.com/article/10.3390/ijms26209967/s1. Figure S1: Mean, median, standard deviation (SD) and width of 95% confidence intervals (CI) of item-response probabilities across 500 bootstrap samples for each latent class. Table S1: Patient characteristics stratified by neutropenia status.

## Abbreviations

The following abbreviations are used in this manuscript:

AIC	Akaike information criterion
ANOVA	Analysis of variance
ARDS	Acute respiratory distress syndrome
aBIC	(Sample size–) adjusted Bayesian information criterion
BIC	Bayesian information criterion
CAR	Chimeric antigen receptor
CI	Confidence interval
CRP	C-reactive protein
CRS	Cytokine release syndrome
DNN	Deep Neural Network
HLH	Hemophagocytic lymphohistiocytosis
HSCT	Hematopoietic stem cell transplantation
ICU	Intensive care unit
IL-6	Interleukin-6
IQR	Interquartile range
LCA	Latent class analysis
LOS	Length of stay
LRT	Likelihood ratio test
MICU	Medical intensive care unit
Npar	Number of estimated parameters
OR	Odds ratio
PCT	Procalcitonin
SAPS	Simplified Acute Physiology Score II
SD	Standard deviation
SLE	Systemic lupus erythematosus
TISS	Therapeutic Intervention Scoring System
XGB	eXtreme Gradient Boosting
