# Supplementary material for: Prediction of Hyperinflammatory Phenotypes in Critically Ill Patients via Routine Clinical Data and IL-6: Towards Personalized Anti-Inflammatory Therapy
Source: Int J Mol Sci. 2025 Oct 13;26(20):9967. doi: 10.3390/ijms26209967 (PMC12562335; doi:10.3390/ijms26209967)
Supplement: Supplementary file 1 [file ijms-26-09967-s001.zip › ijms-3910866-supplementary.pdf]

Supplementary Materials

Prediction of Hyperinflammatory Phenotypes in Critically Ill Patients via Routine Clinical Data and IL-6: Towards Personalized Anti-Inflammatory Therapy

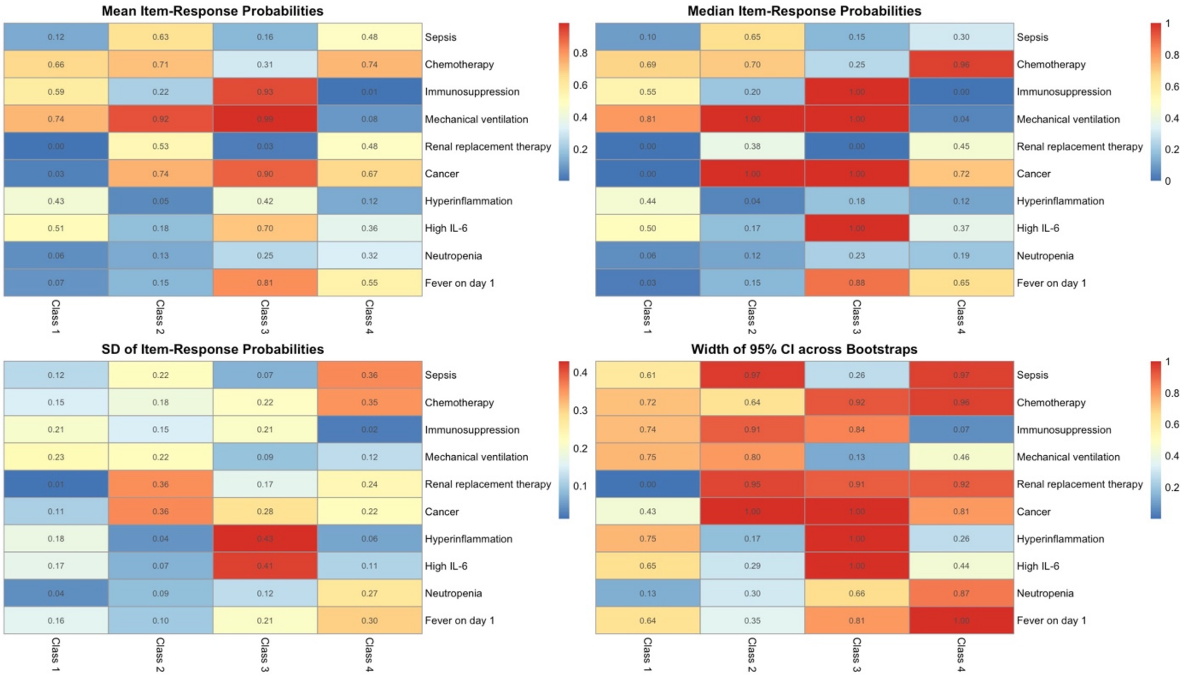

Figure S1. Mean, median, standard deviation (SD) and width of 95% confidence intervals (CI) of item-response probabilities across 500 bootstrap samples for each latent class. Higher values (yellow to red) indicate greater variability and lower stability of item-class assignments, whereas lower SD values (blue) indicate more stable assignments. Items include sepsis, chemotherapy, immunosuppression, mechanical ventilation, renal replacement therapy, cancer, hyperinflammation, high IL-6, neutropenia, and fever on day 1.

Table S1. Patient characteristics stratified by neutropenia status.

Patient Characteristics	No neutropenia[1] N = 129	Neutropenia[1] N = 31	p value[2]
**Sex: female**	38 (29%)	14 (45%)	0.094
**Age** [years]	61 (46, 71)	50 (36, 63)	**0.008**
**Diagnosis**			
Other	88 (68%)	1 (3.2%)	**< 0.001**
Hematologic	24 (19%)	29 (94%)	
Oncologic	17 (13%)	1 (3.2%)	
**Chemotherapy**	14 (11%)	10 (32%)	**0.009**
**Immunosuppression**	21 (16%)	19 (61%)	**< 0.001**
**Autologous HSCT**	2 (1.6%)	3 (9.7%)	0.050
**Allogeneic HSCT**	8 (6.2%)	13 (42%)	**< 0.001**
**Sepsis**	44 (34%)	25 (81%)	**< 0.001**
**Mechanical ventilation**	63 (49%)	22 (71%)	**0.027**
**Renal replacement therapy**	14 (11%)	4 (13%)	0.8
**Noradrenaline day 1** [µg/kg/min]	0 (0, 14)	6 (0, 66)	0.2
**Fever day 1**	28 (22%)	16 (52%)	**< 0.001**
**TISS**	15 (8, 19)	15 (10, 22)	0.3
**SAPS**	44 (31, 62)	58 (39, 74)	**0.019**
**Inflammatory phenotype**			
Hyperinflammatory	47 (36%)	20 (65%)	**0.004**
Non-hyperinflammatory	82 (64%)	11 (35%)	
**IL-6 day 1 [pg/mL]**	130 (35, 684)	7866 (333, 62835)	**< 0.001**
**IL-6 level**			
High	57 (44%)	23 (74%)	**0.003**
Low	72 (56%)	8 (26%)	
**Lactate day 1** [mmol/L]	2.1 (1.3, 3.6)	2.2 (1.2, 4.7)	> 0.9
**Lactate day 2** [mmol/L]	2.20 (1.30, 3.40)	2.10 (1.50, 3.90)	0.8
**CRP day 1** [mg/L]	79 (19, 191)	210 (108, 307)	**< 0.001**
**CRP day 2** [mg/L]	106 (36, 205)	278 (153, 318)	**< 0.001**
**PCT day 1** [ng/mL]	1 (0, 7)	17 (1, 41)	**< 0.001**
**PCT day 2** [n**g/mL]**	2 (0, 16)	30 (2, 148)	**< 0.001**
**Cortisol day 1** [nmol/L]	312 (190, 485)	507 (231, 678)	**0.033**
**Neutrophils** [x109/L]	8 (4, 16)	0 (0, 0)	**< 0.001**
**Leukocytes** [x109/L]	11 (6, 18)	0 (0, 1)	**< 0.001**
**ICU LOS** [days]	5 (3, 13)	8 (4, 20)	**0.048**
**Mortality**	46 (36%)	13 (42%)	0.5

[1] n (%); median (Q1; Q3)

[2] Wilcoxon rank sum test; Pearson's Chi-squared test; Fisher's exact test

HSCT hematopoietic stem cell transplantation, *TISS* therapeutic intervention scoring system, *SAPS* simplified acute physiology score, *IL-6* interleukin-6, *CRP* C-reactive protein, *PCT* procalcitonin, *ICU* intensive care unit, *LOS* length of stay
